# Ether Lipid-Mediated Antioxidant Defense in Alzheimer’s Disease

**DOI:** 10.3390/antiox12020293

**Published:** 2023-01-28

**Authors:** Mariona Jové, Natàlia Mota-Martorell, Èlia Obis, Joaquim Sol, Meritxell Martín-Garí, Isidre Ferrer, Manuel Portero-Otin, Reinald Pamplona

**Affiliations:** 1 Department of Experimental Medicine, Lleida Biomedical Research Institute (IRBLleida), Lleida University (UdL), E-25198 Lleida, Spain; 2 Research Support Unit (USR), Catalan Institute of Health (ICS), Fundació Institut Universitari per a la Recerca en Atenció Primària de Salut Jordi Gol i Gurina (IDIAP JGol), E-25007 Lleida, Spain; 3 Department of Pathology and Experimental Therapeutics, University of Barcelona (UB), E-08907 Barcelona, Spain; 4 Neuropathology Group, Institute of Biomedical Research of Bellvitge (IDIBELL), E-08907 Barcelona, Spain; 5 Network Research Center of Neurodegenerative Diseases (CIBERNED), Instituto Carlos III, E-08907 Barcelona, Spain

**Keywords:** antioxidants, lipidomics, lipid oxidation, human brain, neurodegeneration, plasmalogens

## Abstract

One of the richest tissues in lipid content and diversity of the human body is the brain. The human brain is constitutively highly vulnerable to oxidative stress. This oxidative stress is a determinant in brain aging, as well as in the onset and progression of sporadic (late-onset) Alzheimer’s disease (sAD). Glycerophospholipids are the main lipid category widely distributed in neural cell membranes, with a very significant presence for the ether lipid subclass. Ether lipids have played a key role in the evolution of the human brain compositional specificity and functionality. Ether lipids determine the neural membrane structural and functional properties, membrane trafficking, cell signaling and antioxidant defense mechanisms. Here, we explore the idea that ether lipids actively participate in the pathogenesis of sAD. Firstly, we evaluate the quantitative relevance of ether lipids in the human brain composition, as well as their role in the human brain evolution. Then, we analyze the implications of ether lipids in neural cell physiology, highlighting their inherent antioxidant properties. Finally, we discuss changes in ether lipid content associated with sAD and their physiopathological implications, and propose a mechanism that, as a vicious cycle, explains the potential significance of ether lipids in sAD.

## 1. Introduction

A healthy adult human brain is one of the richest tissues in lipid concentration of the human body, accounting for about 12% of the fresh weight and 50% of the dry matter of the brain [1]. Brain lipids display a great deal of functional and structural diversity. The main lipid categories and classes are present in neurons and glial cells as an expression of the different structural and functional needs related to membrane composition and organization, signaling pathways, and homeostasis of oxidative stress [1,2,3,4]. The human brain accomplishes a broad range of functions, from motor to cognitive, which are dependent on the organization of groups of diverse neuronal and glial cell populations. The expression of specific lipid profiles contributes to the functional and morphological diversity among neuronal and glial cells [5,6,7].

Brain regions/areas display differential vulnerabilities to aging and age-related neurodegenerative processes. Among them may be highlighted the sporadic Alzheimer’s disease (sAD), where aging itself is the main risk factor [4,8,9]. The reason why some individuals age with a well-preserved brain function, whereas others decline and develop sAD, is still not known. We hypothesized that lipid-derived adaptative mechanisms have evolved to maintain homeostasis of the brain oxidative stress and keep neural cells and brain function during an entire lifespan, and that the alteration of this lipid composition leads to or favors the onset and progression of sAD pathology.

Here, we explore the idea that ether lipids, a subclass of glycerophospholipids, actively participate in the pathogenesis of sAD. Firstly, we evaluate the quantitative relevance of the ether lipids in the human brain, as well as their implication in the human brain evolution. Secondly, we analyze the roles of ether lipids in neural cell physiology, highlighting their inherent antioxidant properties. Finally, we evaluate the changes associated with sAD and their consequences, and propose a mechanism that, in a vicious cycle, could explain the significance of ether lipids in sAD.

## 2. Lipid Species and the Human Brain

The whole adult human brain comprises the largest diversity of lipid categories, classes, subclasses and molecular species. For instance, the brain contains a great diversity of glycerophospholipids (GPs) [1], as well as sphingolipids (SPs), with a very significant amount of molecular species [10]. Furthermore, cholesterol and its derivatives are also relevant in the brain, which contains a quarter of the human body total cholesterol [11].

**Figure 1:**
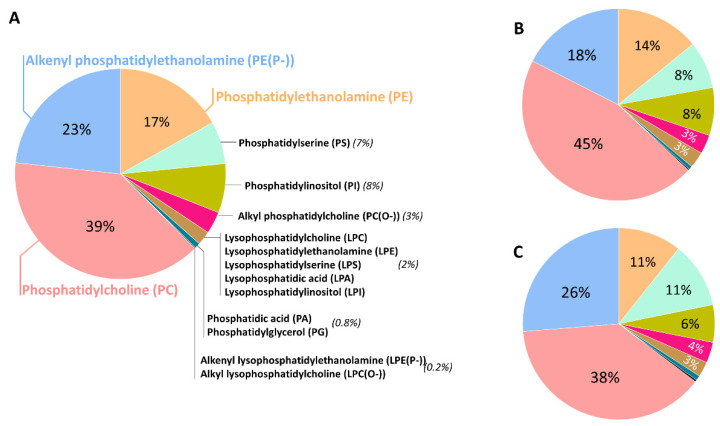
Glycerophospholipid distribution in the human prefrontal cortex (**A**), entorhinal cortex (**B**), and cerebellum (**C**) from healthy adult individuals. Lipidomic analysis was performed in a LC-MS/MS platform. Data obtained from reference [18].

GPs are the main lipid category extensively present in neural cell membranes, with a very significant presence for the ether lipid subclass. In the human brain, GPs represent approximately 5% of the wet weight in the whole brain, 4% of the gray matter (GM) and 7% of the white matter (WM). Diacylglycerophosphates (PAs), a central intermediary in the biosynthesis pathways of both neutral lipids and GPs, occurs in low concentrations in the brain (2% of total GPs). The predominant form of glycerophosphocholines present in the human brain is diacylglycerophosphocholines (PCs) (32.8%), with palmitic acid (16:0) and oleic acid (18:1n-9) as the most representative fatty acid (FA) components [12,13,14,15]. Its ether lipid forms, the 1-(1Z-alkenyl),2-acylglycerophosphocholines (PC plasmalogen, or PC(P-)) and the 1-alkyl,2-acylglycerophosphocholine (PC(O-)), are a minor fraction, representing only 2% of total glycerophosphocholines in the brain. Glycerophosphoethanolamines are quantitatively the main GPs in the human brain (35.6%) [15,16] and the predominant form is the 1-(1Z-alkenyl),2-acylglycerophosphoethanolamines (PE plasmalogen or PE(P-)), accounting for 50–60% of the glycerophosphoethanolamine class. The alkylacyl (PE(O-)) form content is low (3–7%), whereas diacylglycerophosphoethanolamines (PEs) make up the remaining amount of glycerophosphoethanolamines. Their total FA profile indicates a selective positional distribution. Thus, the position-1 of sn-glycerol is occupied mainly by saturated and monounsaturated fatty acids (16:0, stearic acid (18:0), and 18:1n-9), both in the WM and GM; whereas position-2 consists of the polyunsaturated fatty acids (PUFAs), and these are more abundant in the GM than in the WM. The content of glycerophosphoserines in the human brain is approximately 16.6% [15,16] of total GPs. They are mostly present as diacylglycerophosphoserines (PSs, more than 90%) and also as the 1-(1Z-alkenyl),2-acylglycerophosphoserine (PS(P-)), and contain FAs 18:0, 18:1n-9 and docosahexaenoic acid (DHA, 22:6n-3). Inositolphosphoglycerides represent about 2.6% of total GPs in the human brain [12]. Glycerophosphoinositols and glycerophosphoinositols trisphosphates are additional relevant GPs, with only trace amounts of glycerophosphoinositol bisphosphates. Notably, the highest concentrations of glycerophosphoinositols among animal tissues are present in the neural tissue. The main FAs of this class are 18:0 and arachidonic acid (AA, 20:4n-6). Finally, Kahma et al. [17] found 0.2% of GPs as glycerophosphoglycerols (PGs) and 0.1% as glycerophosphoglycerophosphoglycerols (cardiolipins) in the human brain. The latter is mainly located in the brain mitochondria. The main FAs included in this minor, but relevant GP fraction, are 16:0, palmitoleic acid (16:1n-7), 18:0, 18:1n-9, linoleic acid (18:2n-6), linolenic acid (18:3n-3) and 20:4n-6. Figure 1 shows the GP composition of different regions of the adult human brain, highlighting that the ether lipid form represents approximately 20% of total GP.

## 3. Ether Lipids and the Human Brain Evolution

The lipidome is a dynamic system strictly regulated and adapted to cell requirements. The human brain has evolved towards the complexity and structural/functional diversity of neural cells, and these adaptative mechanisms also include cell lipidomes. Effectively, lipidomic analyses have revealed that each human tissue and brain region possess distinctive lipid composition, and that the lipidome signature of the brain is significantly different from that of other non-neural tissues [5]. In particular, from the 5713 detected features, 4727 showed significant differences in the analyzed tissue concentrations, and 75% (3542 features) showed different profiles in the brain compared to non-neural tissues. Notably, these lipidome differences are assigned by specific lipid classes. Thus, the brain lipidome is characterized by an abundance of glycerophosphocholines, glycerophosphoethanolamines, neutral glycosphingolipids, glycerophosphoglycerols and glycosyldiradylglycerols; and a depletion in triacylglycerols, fatty amides and sterols. The enriched lipid species belong to specific lipid subclasses, namely diacylglycerols, dihydroceramides, ceramides and especially 1-(1Z-alkenyl), 2-acylglycerophosphoethanolamines (PE(P-)). Furthermore, within the human brain, the interregional comparison (between cerebellar cortex, primary visual cortex and prefrontal cortex) also showed region-specific differences. Consistent with this observation, additional studies also demonstrated the presence of specific interregional differences in the fatty acid profiles of the human brain [6,7]. Therefore, a general trait of the (human) brain is the high selectivity in lipid classes and subclasses present in their lipidome. The distinctive trait of lipids between the brain and other tissues also suggests that they are a specific adaptation, facilitating the unique structural and functional properties of the cell membranes in the brain tissue.

Other observations about the brain lipidome evolution give additional support to the above-expressed ‘rule’ [5]. Thus, when the human lipidome is compared to that of primates (macaque and chimpanzee) and mammalian (mouse) tissues, the existence of a species-specific and a brain-specific lipidome is corroborated. Lipids systematically distinguish the brain from other tissues for each animal species, suggesting a tissue-specific lipidomic trait conservation across animal species. Additionally, the lipid classes and subclasses that distinguish the brain and other tissues are shared among animal species, indicating a basic compositional specificity of the brain lipidome. Importantly, the magnitude of differences in the lipidome profile between the brain and other tissues increase parallelly with the gain in the brain’s functional capacity from mice to humans. Furthermore, the greatest expression of this change occurs at the level of the human neocortex, and is associated in a specific way with the high concentration of PE(P-) content. Taken together, these findings demonstrate the human brain-specific features, confirming that the brain lipid composition evolves rapidly, and suggesting that lipids, and especially ether lipids, played a key role in the evolution of brain functionality.

## 4. Basic Traits of Ether Lipids: Structure, Metabolism, and Function

Contrary to conventional GPs that have acyl chains joined by ester bonds, in both sn-1 and sn-2 positions, ether lipids have an alkyl chain linked by an ether bond in the sn-1 position. Specifically, ether lipids can contain both alkyl (1-O-alkyl, plasmanyl) and alkenyl (1-O-alk-1′-enyl, plasmenyl) residues (for review, see [19,20]). The “plasmenyl” forms are also known as plasmalogens, and they were described for first time in 1924 by Feulgen and Voit [21]. Plasmalogens are the most common form of ether lipids. The alkyl–ether linkage is represented by the “O-” prefix, and the (1Z)-alkenyl ether (plasmalogen) species by the “P-” prefix. The alkyl/alkenyl residues ―usually palmitoyl, stearyl, and oleyl alcohols― are mainly located in the sn-1 position; whereas the sn-2 position is usually substituted by PUFAs, such as 20:4n-6 and 22:6n-3. In the human brain, ether lipids mainly belong to the lipid class PE, in a lesser degree to PC and 1-O-alkyl-2-acylglycerols (alkyl-DG), and occasionally to PSs or PIs (see also Figure 1).

**Figure 2:**
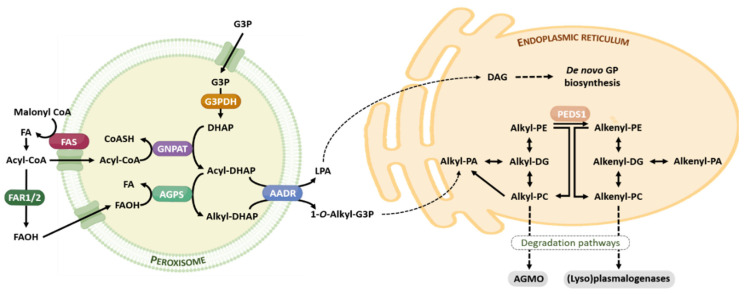
Ether lipid metabolism. The ether lipid biosynthesis process initiates in peroxisomes and is subsequently completed in the endoplasmic reticulum. Briefly, the early steps of ether lipid biosynthesis in the peroxisomes are based on substrates derived from fatty acid metabolism (AcylCoA) and glycolysis (DHAP), and the rate-limiting step is the provision of FAOH. The completion of GP biosynthesis occurs in the endoplasmic reticulum using DAG and Alkyl-PA as precursor molecules. The catabolism of ether lipids proceeds by their lyso-forms for alkyl lipids, and is catalyzed by AGMO, and by (lyso)plasmalogenases in the case of alkenyl lipids. Abbreviations: AAG, alkyl-acylglycerol; AGMO, alkylglycerol monooxygenase; AGPS, alkylglycerone phosphate synthase; DAG, diacylglycerol; DHAP, dihydroxyacetone phosphate; FAOH, fatty alcohol, FAR 1 and FAR2, fatty acyl-CoA reductase; FAS, fatty acid synthase; G3P, glycerol 3-phosphate; G3PDH, glycerol 3-phosphate dehydrogenase; GNPAT, glyceronephosphate O-acyltransferase; GP, glycerophospholipid; LPA, lysophosphatidic acid; PEDS1, plasmanylethanolamine desaturase. For additional details see references [19,20].

The ether lipid biosynthesis initiates in the peroxisome and is completed in the endoplasmic reticulum. Its synthesis is regulated by a feedback mechanism, as a result of sensing the content of ether lipids (in particular, plasmalogens) and/or its metabolites at the membrane level [22]. Analogously to the brain cholesterol content, the brain has the capacity to tightly self-regulate their plasmalogen content, which is independent of circulating plasmalogen and its fluxes, and the transport through the blood-brain barrier [22]. Ether lipids have a short half-life, between 30 min and 3 h. Figure 2 offers a brief summary of the ether lipid biosynthesis and degradation pathways.

Although the full functional spectrum of ether lipids remains to be elucidated, we currently know that they are involved in a variety of biological functions in the brain tissue, including structural roles, membrane trafficking, cell signaling and oxidative-stress homeostasis [23].

### 4.1. Structural Roles

Ether lipids are structural components of cell membranes and the subcellular compartments. The presence of an ether bond in the phospholipid structure provokes a conformational change, which produces a tighter packing of these lipids and alters the physical properties of the membranes. These properties facilitate a stronger intermolecular hydrogen bonding between the headgroups [24], promote close alignment [25,26] and decrease membrane fluidity. The importance of this distribution in structures such as myelin, is confirmed by the abundance of myelin in plasmalogens [27] and by the observation that ether lipid deficiency in both mouse models and human subjects often presents defects in myelination [28].

Another important observation is the high concentration of plasmalogens in lipid raft microdomains [29,30]. In line with this, the plasmalogen-deficient GNPAT knockout mice show aberrant lipid raft formation, along with alterations in cholesterol location [30].

### 4.2. Membrane Trafficking

An inverted hexagonal structure of the cell membranes is related to membrane fusion [31]. Ether lipids, especially plasmalogens, have inherent properties that affects membrane geometry. Specifically, plasmalogen-enriched membranes have a marked tendency to form non-lamellar, inverse hexagonal structures [32], thus facilitating the processes of membrane trafficking, which is particularly relevant, for instance, at a synaptic level and, consequently, neurotransmission. Effectively, the membrane of synapses, as well as synaptic vesicles, show a richness in ether lipids. In line with this, it has been previously described that the synapsis process is impaired in ether lipid-deficient mice [33]. Furthermore, plasmalogens are important components of exosomes [23], but their relevance for the brain function is currently unknown.

### 4.3. Cell Signaling

Ether lipids are a source of a wide spectrum of signaling mediators [23], some of which have not even been described at the brain level. Thus, the list of ether lipids and derivatives involved in signaling includes, for instance, compounds such as alkylglycerol, alkyl-lysophosphatidic acid (alkyl-LPA), alkenyl-LPA, ether-linked diglycerides, 2-halo fatty aldehydes, lysoplasmalogen, lyso-PAF, N-acyl ethanolamine plasmalogen (pNAPE), plasmalogens, platelet-activating factor (PAF), plasmanyl phospholipids and GPI anchor [23]. These ether lipid compounds have demonstrated to interact with components related to diverse signaling pathways such as AKT/PKB, PKC, PPAR, LXR, GPCR and MAPK [23]. As derived mostly from in vitro and animal models studies, these pathways are potentially involved in different neuronal and/or glial cell processes, such as energy metabolism, myelination, neurotransmission (synaptic plasticity), pro- and anti-inflammatory responses, cholesterol homeostasis and oxidative stress. However, the relevance of these compounds and signaling pathways in the human brain physiology and sAD still is thus far incomplete.

Due to the preferential presence of PUFA in the sn-2 position, ether lipids have also been proposed as a second-messenger precursor reservoir [34]. Among these PUFAs, 20:4n-6 and 22:6n-3 (DHA) must be highlighted due to their particular biological and physiological importance as precursors of eicosanoids and docosanoids, respectively. Interestingly, it has been suggested that the participation of DHA in diverse molecular events relates to synaptic plasticity, neuro- and synaptogenesis, neurite outgrowth and learning and memory-related processes, as well as neuroprotective antioxidant mechanisms [35,36]. Plasmalogens also act as a reservoir for AA. AA has been involved in both physiological (synaptic plasticity) and physiopathological (sAD) processes [37].

## 5. Plasmalogens as Endogenous Antioxidants in the Human Brain

The appearance and use of ether lipids (plasmalogen) by eukaryotic cells is directly related to the origin of the aerobic life [38] and the subsequent generation of free radicals (reactive oxygen species, ROS), which demanded the incorporation of antioxidant defense mechanisms to ensure cell survival. The biosynthesis pathway of plasmalogen in eukaryotic cells is accomplished using an oxidative mechanism that, similarly to the aerobic desaturation of fatty acids, needs a source of molecular oxygen. The result is the generation and incorporation of a vinyl ether bond in the plasmalogen structure which confers to its special properties. One of these properties is that this kind of bond confers a high ROS sensitivity to plasmalogens, generated physiologically during oxidative metabolism by cells. Thus, the oxidative metabolism that is needed for plasmalogen synthesis results in a molecule that, in turn, is sensitive to oxidative damage. This oxygen sensitivity of plasmalogens was described in 1972 [39]. Therefore, it may be suggested that plasmalogens, as targets of ROS, act as free radical scavengers [40,41,42,43,44] and may be considered as a potential endogenous antioxidant mechanism inside lipid membranes. In line with this, the rich plasmalogen content observed in the brain [5] may be interpreted as an additional adaptive response to the high oxidative conditions present in the human brain [45,46], while protecting unsaturated membrane lipids from oxidation by free radicals [44]. Consistent with this concept, plasmalogen-deficient animal and cell models are more prone to oxidative damage than control models [40,47,48,49]. In the presence of ROS, plasmalogens are easily degraded with scission at the alkenyl ether bond [40]. In this situation, cells have the ability to acylate the resulting 2-monoacyl-glycerophosphatidylethanolamine, with the subsequent formation of diacylglycerophosphatidylethanoalmine, or to deacylate the resulting lysophospholipid.

## 6. Ether Lipids in Alzheimer’s Disease

Human inherited peroxisomal disorders that cause ether lipid deficiency seriously compromises human health [35]. Recent studies suggest that changes in ether lipid content are also associated with sAD. Based on the current evidence, brain aging and sAD may be considered as (textually) ‘(i) a multifactorial and progressive neurodegenerative biological process, (ii) characterized by the early appearance of 3R + 4Rtau NFTs (neurofibrillary tangles), (iii) later deposition of β-amyloid and SPs (senile plasques), (iv) with particular non-overlapped regional distribution of NFTs and SPs, (v) which are preceded by, and occurring in parallel with, molecular changes involving determining subcellular structures and functions; (vi) accompanied by progressive neuron loss and brain atrophy, (vii) prevalent in human brain aging, and (viii) manifested as pre-clinical AD, and progressing not universally to mild cognitive impairment due to AD (MCI-AD), and mild, moderate, and severe AD dementia (ADD)’ [50]. Currently, sAD is the most prevalent neurodegenerative disease and cause of dementia worldwide. sAD is estimated to affect more than 35 million people, with a robust rise in the number of patients predicted [51,52]. Considering the preeminent role of ether lipids in brain function, it can be postulated that potential changes in the content and derived functions from ether lipids could play a mechanistic role in the onset and progression of sAD.

### 6.1. Ether Lipids and Alzheimer’s Disease

Despite the differences in the brain regions examined, disease stages, comorbidity and analytical methods applied, results systematically converge into the concept that, in the regions specifically affected by this pathological condition, sAD is associated with lower levels of PE(P-) and PC(P-) in postmortem brain samples (Table 1). Importantly, decreased plasmalogen levels seem to be associated, to a lesser extent, with the physiological aging process [12,53], which is perfectly compatible with the fact that sAD is an age-related disease, and the existence of a continuum between aging and sAD [50]. In sAD, the deficit is present in WM, where it appears very early in the natural history of the disease, as well as in GM, where it correlates with neuropathological staging and cognitive decline (Table 1). A significant number of studies also state a decrease in the content of particular plasmalogen molecular species in the plasma/serum of sAD patients (see Table 1), suggesting their utility as a potential biomarker for cognitive decline. The detection of specific plasmalogen species might be valuable as components of a biomarker panel, but more studies are needed to validate this scenario.

The origin of plasmalogen depletion in sAD is an unsolved issue. However, the most plausible cause is the preferential affectation of these lipids under oxidative-stress conditions, a scenario clearly increased in sAD [79,80]. Compatible with this observation, a peroxisomal dysfunction, induced by aging and by sAD pathology, could also lead to impaired plasmalogen biosynthesis [61,81]. Another factor involved in plasmalogen depletion in sAD could proceed from a detrimental role of amyloid-beta, either by activation of plasmalogen-selective phospholipase A2 [82] or by dysfunction of the ADHAPS protein, thus limiting plasmalogen biosynthesis [59]. Other authors suggest that the increased activity of plasmalogen-selective phospholipase A2 favors the production of lysoplasmalogens, resulting in excessive vesicular fusion, and eventually causing synaptic failure [83]. Furthermore, signaling alterations resulting from plasmalogen reduction―such as those described in the ERK and AKT pathways―may cause additional damage through the characteristic hyperphosphorylation of the tau protein observed in sAD [84,85,86]. Plasmalogens may also have a direct effect on the generation of amyloid-beta peptides. Cleavage of APP has been observed to take place in lipid rafts [87]. Therefore, the potential alteration of these membrane domains caused by the depletion of plasmalogens may also influence the production of amyloid-beta peptides. DHA deficits may also play a role in sAD, as described [4]. Indeed, ether lipid deficiency also causes a decline in the brain DHA levels. Therefore, these observations suggest that the decrease in ether lipids in sAD also participates in the pathogenic processes via DHA depletion [4,88,89]. Alternatively, based on the neuroinflammatory condition described in sAD [50] and results obtained in AD mouse models, a recent study claims that the inflammatory response downregulates Gnpat expression via NF-jB signaling and c-Myc binding to the Gnpat promoter [84].

In any case, these findings seem to suggest that the plasmalogen deficits may be a consequence of increased oxidative stress present in sAD, but, in turn, play a causative role in the pathogenic process, aggravating the disease progression. Aligned with the proposed role of plasmalogens as ROS scavengers, their depletion might amplify the oxidative stress burden in the sAD brain. However, more studies are needed to establish the role of ether lipids (and especially plasmalogens) in the modulation of membrane function, membrane stabilization/destabilization, signal transduction, and oxidative stress in AD animal models and sAD patients.

### 6.2. Potential Interventions to Ameliorate the Ether Lipid Content in the Brain Tissue

Treatment approaches targeting ether lipid deficiency in AD are limited. Two therapeutic lipid-based approaches for managing AD have been attempted to achieve plasmalogen replacement therapy: oral administration and liposome (or exosome-like liposomes) delivery (reviewed in [23,35,90]). These approaches seems to have promising results in replacing ether lipids in non-neural tissues, while the results have been unsuccessful at the brain level [23,35,90]. For the first approach, oral administration, treatment with alkylglycerols, is a good candidate because their ability to restore plasmalogen content is well-documented in ether lipid-deficient cultured cells. However, studies in wild-type rats indicated that alkylglycerol replaces plasmalogens in various non-neural tissues, but not in the brain. This observation was later reaffirmed in a systematic preclinical trial in the experimental model Pex7 KO mice, which confirmed that, upon oral batyl-alcohol (an alkylglycerol) treatment, the levels of plasmalogens are reestablished in diverse organs, but not in the brain. These results and other studies infer that brain tissue relies exclusively on its own ether lipid biosynthesis and does not incorporate exogenous ether lipids, and that plasmalogens and their precursors cannot efficiently cross the blood-brain barrier (BBB). Thus, how orally-administered plasmalogens would impact brain function currently remains unclear. Therefore, generating synthetic plasmalogen precursors able to overcome the BBB, or developing novel delivery systems, is needed. For the second approach, based on the use of liposomes or exosome-like liposomes, studies are very preliminary and far from conclusive. However, improving the oxidative conditions existing in the human brain in AD should be a priority that would likely also impact the optimization of the ether lipid content. Indeed, interventions such as heterochronic parabiosis, exercise and caloric restriction, and treatments with endogenous lipid mediators, such as palmitoylethanolamide, that have demonstrated their capacity to modulate oxidative stress and neuroinflammation, have also demonstrated that aging-associated cognitive, cellular and molecular impairments can be restored to more youthful levels [91,92]. Therefore, it is also possible to postulate potential beneficial effects for sAD. However, more studies are needed to validate this idea.

## 7. Conclusions

**Figure 3:**
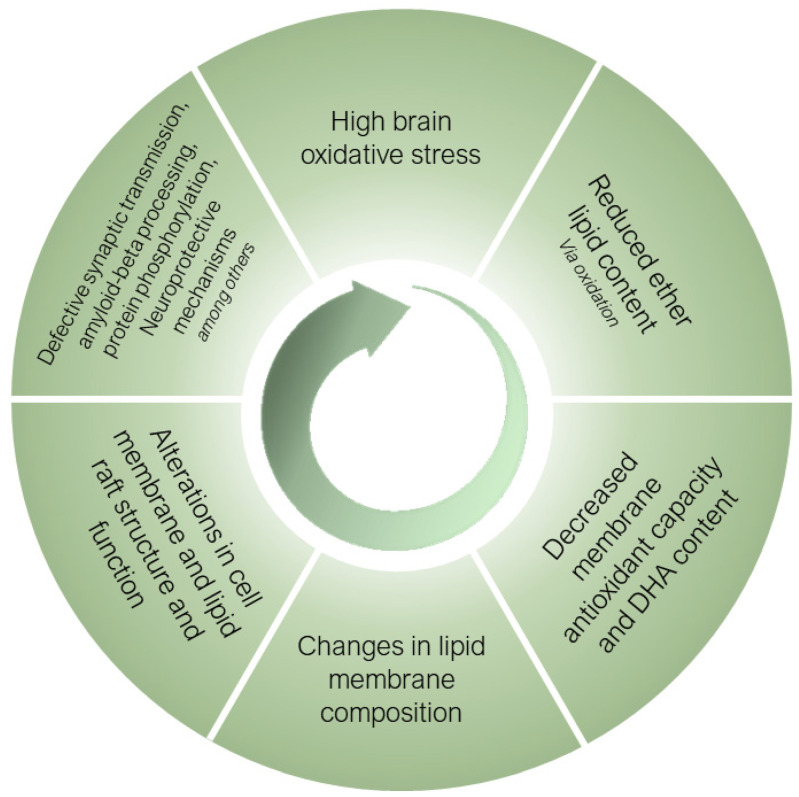
Mechanistic pathway for the potential role of ether lipids in the pathogenesis of sporadic Alzheimer’s disease. For an explanation, see main text.

Ether lipids, especially submitted to a selective process during the human brain evolution, are essential structural components of neural cell membranes. Their inherent properties, derived from their unique architecture, allow for neural cells to perform a wide range of specialized functions, while protecting membranes from the high oxidative stress conditions present in the human brain and exacerbated in sAD. Figure 3 proposes a mechanistic pathway for the potential role of ether lipids in the pathogenesis of sAD. Thus, the most aggressive oxidative stress conditions existing in sAD induces the oxidative damage of ether lipids, as preferential targets at the membrane level, causing a decrease in their content with a concomitant loss of PUFA (especially DHA) present in its structure, leading to a loss of antioxidant and neuroprotective capacity. These changes result in alterations in the composition of the neural cell membranes, with the subsequent effects at the structural level, as well as of the lipid rafts. The negative consequences are multiple defects which alter the processing of amyloid-beta, the vesicular traffic at the synaptic level, protein hyperphosphorylation and neuroprotective mechanisms, among others. These alterations worsen the homeostasis of oxidative stress and, as a vicious cycle, the system feeds itself in a detrimental spiral that favors the onset and progression of AD. For the future, the development of ether lipid precursors that could cross the blood-brain barrier and alter the clinical course of sAD or its appearance, would be of great interest. However, improving the oxidative conditions existing in the human brain in AD should be a primary objective that would likely also impact the optimization of the ether lipid content.

## Figures and Tables

**Table 1:** Updated list of studies analyzing ether lipid content in Alzheimer’s disease (AD).

Tissue	Analytical Approach	Cases	Effect of AD on Ether Lipid Content	Reference
**Human Brain Tissue**				
Temporal cortex, cerebellum, caudate nuclei, substantia nigra	TLC	Ctl (9) vs. AD (9).	Decreased plasmalogens in TC, not in cerebellum, caudate nuclei and substantia nigra.	[54]
Frontal cortex, hippocampus, white matter	HPLC and GC	Ctl (13) vs. AD (15).	Decreased PE-plasmalogens (PE(P-)).	[55]
Frontal cortex, parietal cortex, temporal cortex and cerebellum (grey matter and white matter)	ESI/MS	Subjects with a spectrum of AD clinical dementia rating (CDR from 0 to 3) (*n* = 6 per group).	Decreased PE(P-) with increase in CDR, except in cerebellum.	[56]
Superior/middle frontal, Inferior parietal occipital, superior temporal, cerebellum	NMR	Ctl (6–16) vs. AD (37–43) Most of the control subjects presented concomitant neuropathological disorders.	Increased PE(P-) in frontal and temporal cortex.	[57]
Frontal cortex	TLC	Ctl (26) vs. AD (39).	Decreased PE(P-).	[58]
Frontal cortex, temporal cortex, cerebellum	LC-MS	Ctl (14) vs. AD (30).	Decreased PC(P-).	[59]
Prefrontal cortex	TLC	Ctl (9) vs. AD (10).	Decreased PE(P-).	[60]
Gyrus frontalis	GC/MS	Human AD grouped by Braak staging of neuropathology.	Decreased PE(P-).	[61]
Prefrontal cortex, entorhinal cortex, cerebellum	HPLC-QQQ/Ion Trap-MS	Ctl (10) vs. AD (10) per region. AD was defined as “high probability of AD” based on NIA_RI (National Institutes of Health-Reagan Institute) criteria.	No significant differences in any brain region for plasmalogen content.	[18]
Grey matter, white matter	HR-ESI-MS	Young dementia, old dementia and MCI cohort.	Decreased PE(P-) in grey matter from young dementia and old dementia.	[62]
Prefrontal cortex	UPLC-MS/MS	Ctl (20) vs AD (21).	Decreased PC plasmalogens (PC(P-)) species containing 18:0 and 22:6.	[63]
Brain-derived extracellular vesicles (BDEV) from frontal cortex	nESI-UHRAMS and HCD-MS/MS	Ctl (8) vs AD (8).	Increased specific species of PE(P-).	[64]
Frontal cortex (white matter and grey matter)	UPLC-ESI-QTOF-MS/MS	Middle-aged cases (Ctl group, 6) vs. sAD cases (lacking co-morbidities and concomitant brain pathologies) categorized according to Braak and Braak neurofibrillary tangle (NFT) and β-amyloid stages as ADI–II/0-A (*n* = 7); ADIII– IV/0-C (*n* = 5), and ADV–VI/B-C (*n* = 6)	Decreased specific PC(O-), PC(P-) and PE(P-) species in grey matter and white matter with AD progression.	[65]
Frontal cortex	HR-ESI-MS	Ctl (20) vs. MCI (19), EOAD (17), and LOAD (17).	Decreased serine ether GPs (PS(O-), PS(P-)).	[66]
Human Plasma/Serum Tissue				
Serum	LC-MS/MS	>350 non-demented subjects vs. >400 demented subjects (dementia of the Alzheimer’s type).	Decreased PE(P-) 16:0/22:6 with severity pf dementia.	[67]
Serum	LC-MS	Ctl (66) vs. AD patients with an ADS-Cog score between 20 and 46 (40).	Decreased PE(P-) and there is a correlation between plasmalogen depletion and cognitive decline.	[68]
Serum	UPLC-MS	Ctl (46), MCI (143), AD (47).	Decreased ether GPs (PC(O-)).	[69]
Plasma	UPLC-ESI-QTOF-MS and LC-MS/MS	Five hundred and twenty-five community-dwelling participants aged 70 and older and otherwise healthy into this 5 years observational study. Three groups were defined: normal control, aMCI/AD, and converted.	Decreased PC(O-40:6) predict phenoconvertion to either MCI or AD within a 2–3-year timeframe.	[70]
Plasma	MRM-SID-MS	Ctl (73) vs. phenoconverters (28).	Decreased specific PC(P-) species allow to determine the risk of phenoconversion from normal cognition to aMCI or AD.	[71]
Serum	LC using radioactive iodine	NE (cognitively normal elderly) (107) vs. MCOs (memory clinic outpatients) (55).	Decreased PE(P-)	[72]
Plasma	LC-MS	Ctl vs AD.	Decreased PE(P-), bearing the DHA moiety.	[73]
Serum	UPLC-QQQ-MS/MS	Ctl (199), MCI (356), AD (175).	Increased specific ether-linked PCs in earliest AD.	[74]
Plasma	UFLC-MS/MS	From 606 participants, two groups are generated: aged controls and probable AD after 30, 60 and 90 months (ctl vs. aging and phenoconversion to AD).	Increased lysoPAF and PC(P-).	[75]
Plasma	HILIC-ESI-IT-TOF-MS	Ctl (41) vs. Cognitive Impairment (41).	Decreased PE(P-), especially with PUFAs.	[76]
Plasma	HPLC-QQQ-MS	Two cohorts: 1) Ctl (768), MCI (131), AD (211); 2) Ctl (200), MCI (400), mild AD (200).	Decreased PE(O-) and PE(P-) species.	[77]
Plasma	LC ESI-QQQ MS/MS	Preclinical and prodromal AD cases from the ADNI cohort (529 participants).	Decreased content of ether-linked GPs.	[78]

Abbreviations: AD, Alzheimer’s disease; ADAS-cog, Alzheimer Disease Assessment-Cognitive; ADNI, Alzheimer’s Disease Neuroimaging Initiative database; GP, glycerophospholipids; Ctl, healthy control group; CDR, clinical dementia rating; MCI, mild cognitive impairment; EOAD, early-onset Alzheimer’s disease; LOAD, late-onset Alzheimer’s disease; UPLC-ESI-QTOF-MS/MS, ultra-performance liquid chromatography electrospray ionization quadrupole time of flight-tandem mass spectrometry; LC ESI-QQQ MS/MS, liquid chromatography electrospray ionization triple-quadrupole tandem mass spectrometry; HR-ESI-MS, high-resolution electrospray mass spectrometry; GC, gas chromatography; HPLC, high-performance liquid chromatography; TLC, thin-layer chromatography; UPLC, ultra-performance liquid chromatography; MS/MS, tandem mass spectrometry; nESI-UHRAMS and HCD-MS/MS, direct-infusion nano-electrospray ionization (nESI)—ultrahigh resolution and accurate mass spectrometry (UHRAMS) and higher energy collision induced dissociation tandem mass spectrometry (HCD-MS/MS) shotgun lipidome analysis; HPLC-QQQ-MS, high-performance liquid chromatography- triple-quadrupole mass spectrometry; LC-MS/MS, liquid chromatography tandem mass spectrometry; GC/MS, gas chromatography mass spectrometry; NMR, nuclear magnetic resonance; UFLC-MS/MS, ultra-fast liquid chromatography tandem mass spectrometry; HILIC-ESI-IT-TOF-MS, LC-MS method based on the hydrophilic interaction liquid chromatography electrospray ionization-ion trap-time of flight-mass spectrometry; MRM-SID-MS, multiple reaction-monitoring stable isotope-dilution mass spectrometry.
